# Effects of HDL Structure and Function in Peripheral Artery Disease

**DOI:** 10.3390/biom15101419

**Published:** 2025-10-06

**Authors:** Yu-Huang Liao, Semon Wu, Yu-Lin Ko, Ming-Sheng Teng

**Affiliations:** 1 Division of Endocrinology and Metabolism, Department of Internal Medicine, Taipei Tzu Chi Hospital, Buddhist Tzu Chi Medical Foundation, New Taipei City 231016, Taiwan; lyuh5398@gmail.com; 2 Department of Life Science, Chinese Culture University, Taipei 111396, Taiwan; semonwu@yahoo.com.tw; 3 Department of Research, Taipei Tzu Chi Hospital, Buddhist Tzu Chi Medical Foundation, New Taipei City 231016, Taiwan; 4 The Division of Cardiology, Department of Internal Medicine and Cardiovascular Center, Taipei Tzu Chi Hospital, Buddhist Tzu Chi Medical Foundation, New Taipei City 231016, Taiwan; 5 School of Medicine, Tzu Chi University, Hualien 970374, Taiwan

**Keywords:** HDL subfractions, Ox-HDL, SNP, PAD

## Abstract

The structure and function of high-density lipoprotein (HDL), rather than its concentration, are more important factors in determining HDL activity. HDL particles (HDL-P) are heterogeneous in their composition, size, and antioxidative function. We investigated the levels of HDL subfractions and oxidized high-density lipoprotein (Ox-HDL) and validated their correlation with genetic determinants underlying peripheral artery disease (PAD). We recruited a PAD population stratified by claudication severity (group I) and critical limb ischemia (group II) according to the Rutherford classification. We found that the level of Ox-HDL was significantly increased with Rutherford classification (group II; *p* = 0.001). Conversely, the levels of high-density lipoprotein cholesterol (HDL-C), HDL-P, and small high-density lipoprotein particles (S-HDL-P) were significantly reduced in group II. Three single nucleotide polymorphisms (SNPs) were differentially associated with HDL particles and Ox-HDL. Briefly, rs117685211 and rs7934858 showed opposing effects, with rs117685211 and rs148877054 being associated with low levels of HDL subfractions; rs148877054 was significantly associated with M and S-HDL-P. Our study indicated the significance of HDL subfractions and Ox-HDL in the pathogenesis of PAD.

## 1. Introduction

Peripheral artery disease (PAD), a type of cardiovascular disease (CVD), is an atherosclerotic condition characterized by decreased blood flow through the lower extremities, leading to a variety of signs and symptoms [1]. Smoking, hypertension, dyslipidemia, diabetes, chronic kidney disease, and family history of PAD have been found to be major PAD risk factors [2]. Most people with PAD are commonly asymptomatic, but they might experience symptoms of claudication, tissue loss, or rest pain. These symptoms may include the clinical expression of chronic limb ischemia, which is correlated with a high risk of limb loss [2], potentially major limb amputation, and increased risk of stroke, myocardial infarction, and mortality [3].

Notably, HDLs are composed of a heterogeneous lipoprotein family comprising subclasses of lipid and protein components of different sizes and shapes. Mature HDLs exhibit constant dynamic remodeling in their 4–5-day lifecycle owing to interactions with several enzymes, such as hepatic and endothelial lipases [4]. HDLs play a major role in scavenging excess cholesterol through reverse cholesterol transport (RCT). The excess cholesterol would be transported to the liver and organs, which have high cholesterol requirements, or be exchanged with low-density lipoprotein cholesterol (LDL-C) for disposal [4]. Despite strong evidence indicating that the level of HDL-C is the major determinant of RCT, Mendelian randomization studies have shown that HDL-C has no definite causal association with cardiovascular benefits, suggesting that genetic variants associated with increased levels of HDL-C do not have an effect on the risk of myocardial infarction [5,6]. Furthermore, a meta-analysis showed that increased levels of HDL-C were not associated with an improvement in cardiovascular outcomes [7].

Recent evidence indicated that the structure and function of HDL, rather than the concentration of HDL-C, are the most important factors determining its activity. HDLs exert their cardioprotective functions owing to their diverse protein and lipid compositions [8]. For instance, dysfunctional HDL, which is defined as loss of the cardioprotective function of HDL, occurs because of changes in the component of HDL particles, such as the quantity and type of proteins and lipids. Therefore, new strategies for the measurement and restoration of HDL functionality [9] that focus on the quality of HDL subfractions rather than HDL-C alone are needed.

The quality of HDLs depends on their dynamic structure and pleiotropic function. Owing to their dynamic structure and associated differing physicochemical properties, HDLs can be separated into several subclasses [10]. Jomard and Osto [11] suggested nuclear magnetic resonance (NMR) as the most convenient method of HDL classification for clinical usage [12] as it enables the measurement of several distinct HDL subfractions [13], namely large HDL-P, medium HDL-P, and small HDL-P, from whole plasma without requiring preliminary isolation. Recent studies have shown that type 2 diabetes mellitus was positively associated with the level of small HDL-P and inversely associated with that of large HDL-P [14], whereas coronary heart disease (CHD) was inversely associated with the levels of small and medium HDL-P [15].

In addition, HDL oxidation affects both protein and lipid components. For instance, the ability of oxidized HDL (Ox-HDL) to promote cellular cholesterol efflux is reduced, thus losing its cardioprotective properties. Apart from RCT, HDLs have several other beneficial functions, including antioxidant capacity, anti-inflammatory effects, stimulation of nitric oxide (NO) production, and antiapoptotic effects [16]. Because of their susceptibility to oxidative modifications by various oxidants, such as various myeloperoxidase (MPO)-generated oxidants [17], oxidative modification of lipid and protein components in HDL particles also contributes to the functional loss of HDL. However, as there is no gold standard for assessing HDL function, the exact role of dysfunctional HDL in the pathogenesis of CVD remains unclear. Notably, HDL function is known to depend on genetic, environmental, and lifestyle factors. Modification of the protein or lipid components of HDL under certain conditions might convert HDL particles from being anti-inflammatory to proinflammatory and proatherogenic by limiting their ability to promote RCT and prevent LDL modification.

Several genetic studies have elucidated the mechanism and causal association in the development of complex disorders, such as type 2 diabetes (T2D) and coronary artery disease [18,19]. However, few genetic association studies on PAD have been reported [20,21,22,23,24], including in Taiwanese and other Asian populations. Three study SNPs were chosen from our previous Genome-wide association studies (GWAS) since the sample size is too small for GWAS, and the significant *p* value (10^−5^) of these SNPs did not satisfy the GWAS threshold (*p* < 10^−8^). Therefore, we performed a candidate SNP study of Ox-HDL and HDL subfractions in this population. We thus focused on investigating the levels of HDL subfractions and Ox-HDL and validated the genetic determinants underlying the development of PAD, which might unveil risk factors that allow for early disease detection, improved treatment, and potential prevention of progressive disease.

## 2. Materials and Methods

### 2.1. Study Population

Participants were eligible for inclusion if they had a diagnosis of PAD. Exclusion criteria were age younger than 20 years, pregnancy, and the presence of major illnesses, including malignancies. Ultimately, 80 consecutive hospitalized patients with PAD who underwent endovascular intervention (EVI) and had no known history of malignancy were enrolled in the study from May 2011 to October 2012 at Taipei Tzu Chi Hospital. The PAD lesions in these patients all demonstrated more than 70% diameter stenosis at the lower limbs with either advanced symptoms or critical limb ischemia (CLI) according to the Rutherford classification. CLI of the lower extremities refers to a condition characterized by chronic ischemic at-rest pain, ulcers, or gangrene in one or both legs attributable to objectively proven PAD. The Ethics Committee of the Taipei Tzu Chi General Hospital approved the study.

### 2.2. Laboratory Examination

All participants underwent an initial screening assessment that included obtaining a medical history, recording of vital signs, and measurement of total cholesterol, HDL cholesterol, LDL cholesterol, triglyceride, and fasting plasma glucose, all of which were performed in a central laboratory as previously reported [25,26] before initiating the study. For analysis, 20 mL of venous blood was obtained by vein puncture the morning before breakfast after overnight (8–12 h) fasting. Venous blood samples were drawn from an antecubital vein using a 21-gauge (21G) needle without venous stasis. Following addition of sodium heparin and centrifugation at 3000× *g* for 15 min at 4 °C, serum and plasma samples were obtained. Immediately after centrifugation, plasma samples were frozen and stored at −80 °C prior to analysis.

### 2.3. NMR Lipoprotein Subfractions Analysis

Lipid and Apolipoprotein A1 (ApoA1) measure was quantified by high-throughput nuclear magnetic resonance (NMR) metabolomics (Nightingale Health Ltd., Helsinki, Finland; biomarker quantification version 2020). This platform provides simultaneous quantification of routine lipids, lipoprotein subclass profiling with lipid concentrations within 14 subclasses, fatty acid composition, and various low-molecular-weight metabolites including amino acids, ketone bodies, and gluconeogenesis-related metabolites in molar concentration units. NMR metabolomics was conducted for all plasma samples available in this cohort. Biomarkers were quantified independently for each sample without using information from other samples in the same plate well or same cohort. The average success rate of metabolite quantification was 95%. Technological details and epidemiological applications of the Nightingale NMR platform have been reviewed previously [27,28].

### 2.4. The Measurement of Levels of Oxidized HDL Using an Oxidized HDL ELISA Kit

The OxiSelect™ Human Oxidized HDL ELISA Kit (CML-HDL quantitation; CELL BIOLABS, INC., San Diego, CA, USA) was used to measure the levels of Ox-HDL in plasma samples. Glycoxidation of apolipoprotein A1 leads to the formation of advanced glycation end-product adducts, such as N^ε^-carboxymethyllysine (CML). This oxidized HDL assay kit was designed to selectively quantify CML-modified HDL and has a detection sensitivity limit of <1 ng/mL. Briefly, 20 μL of plasma was transferred to an Eppendorf tube, in which 5 μL of Precipitation Solution 1 and 10 μL of Precipitation Solution 2 were added and mixed well, followed by incubation at 25 °C for 5 min. The mixture was then centrifuged at 6000× *g* and 4 °C for 10 min, and the supernatant was carefully collected and transferred to a new Eppendorf tube. Subsequently, 12 μL of Precipitation Solution 3 and 30 μL of Precipitation Solution 2 were added to the supernatant and mixed well, followed by incubation at 25 °C for 2 h and centrifugation at 18,000–20,000× *g* and 4 °C for 30 min. The supernatant was discarded, and the pellet was resuspended in 100 μL of resuspension buffer, followed by thorough mixing by pipetting up and down. Subsequently, the mixture was centrifuged at 6000× *g* and 4 °C for 10 min, the supernatant was discarded, and the pellet was resuspended in 120 μL 1× Wash solution. The mixture was shaken for 30 min at 4 °C and then centrifuged again at 6000× *g* and 4 °C for 10 min. Finally, the supernatant was transferred to a new tube and stored at 4 °C before performing the ELISA on the same day.

### 2.5. DNA Isolation and Genotyping

Peripheral venous blood was collected from patients and processed the same day. Blood was centrifuged at 3000 rpm for 10 min at 4 °C to separate serum and cells. DNA was extracted as reported previously [29]. Briefly, the buffy coat containing peripheral blood mononuclear cells (PBMCs) was extracted and washed with red blood cell (RBC) lysis. Samples were mixed with a cell lysis buffer for several days after lysing the RBCs in lysis buffer. Proteins were precipitated using a protein precipitation solution. Finally, 95% isopropanol and 80% alcohol were used to isolate total genomic DNA. DNA samples were genotyped using TaqMan SNP Genotyping Assays from Applied Biosystems (ABI; Foster City, CA, USA). The minor allele frequency of 3 SNPs (rs117685211, rs7934858, and rs148877054) are 0.075, 0.21 and 0.045, respectively. No significant deviations from the Hardy–Weinberg equilibrium were detected for these polymorphisms (*p* = 0.658, 0.819, and 0.163 for SNPs rs117685211, rs7934858, and rs148877054, respectively) (Supplementary Table S1).

### 2.6. Statistical Analysis

The clinical characteristics of continuous variables are expressed as the means ± standard deviations, which were tested using a two-sample *t*-test. A chi-square test was used to examine differences in the distribution of categorical data. Levels of Ox-HDL, HDL subfraction, and lipid profile were logarithmically transformed to adhere to normality assumption. A linear regression model was used to analyze Ox-HDL, HDL subfraction, and lipid profile levels in association with the genotypes and confounders. The genetic effect was adjusted for age, sex, BMI, smoking status, and lipid-lowering and antihypertension medication. A post hoc power analysis was performed to evaluate whether the sample size was sufficient to detect statistically significant associations.

## 3. Results

### 3.1. Baseline Data of PAD Population

We recruited a PAD population comprising 46 men and 34 women. Demographic data, lipid profile, and Rutherford classification of participants stratified by the severity of claudication (group I: Rutherford category = 3) and critical limb ischemia (group II: Rutherford category ≥ 4) are summarized in Table 1**.** We found significant differences in the levels of HDL-C and ApoA1 between group I and group II (*p* = 0.018 and *p* = 0.036, respectively). In addition, the percentage of current smokers is significantly higher in group I (*p* = 0.003).

### 3.2. Association of Rutherford Classification with HDL Subfractions, ApoA1, and Ox-HDL

To evaluate the association between the function and structure of HDL and critical limb ischemia in PAD, we analyzed the levels of HDL-P subfractions, ApoA1, and Ox-HDL. We found that the level of Ox-HDL was significantly increased with the severity of PAD classified according to the Rutherford classification (*p* = 0.001). Furthermore, we detected that the level of Ox-HDL was also increased in group II (*p* = 0.001). Conversely, we observed that the levels of HDL-C, ApoA1, HDL-P, and S-HDL-P were significantly reduced in group II (*p* = 0.018, 0.036, 0.012, and 0.01, respectively) (Table 2).

### 3.3. Correlation Between ApoA1, HDL Subfractions, and Ox-HDL

The Pearson correlation analysis indicated that the levels of HDL-C, ApoA1, HDL-P, and S-HDL-P were inversely correlated with the level of Ox-HDL (*p* = 0.001, 0.005, 0.002, and 0.012, respectively) (Table 3).

### 3.4. Study of Association of Candidate SNPs with Levels of HDL Subfraction, ApoA1, and Ox-HDL in PAD Population

To determine the genetic effects on the HDL subfractions, ApoA1, and Ox-HDL, we genotyped several Asian-specific SNPs and analyzed the levels of HDL subfractions and Ox-HDL in the respective samples (Table 4). After adjusting for age, sex, smoking status, and use of lipid-lowering and antihypertensive medications, we determined that participants carrying the minor alleles of the rs117685211 genotype exhibited a significant trend of higher levels of Ox-HDL in the additive and dominant models (*p* = 0.012 and 0.002, respectively), and conversely showed a significant trend of lower levels of ApoA1 and HDL-P (including L, M, and S-HDL-P; all *p* values were ≤0.001 in the additive and dominant models). In contrast, we found that participants carrying the minor alleles of the rs7934858 genotype tended to have a significantly lower level of Ox-HDL in the additive and dominant models (*p* = 0.001 and 0.002, respectively). Furthermore, we observed that the participants carrying the minor alleles of the rs148877054 genotype exhibited a significant trend of lower levels of HDL-P (only presented in the M and S-HDL-P in the additive and dominant models; all *p* values were ≤0.001) and ApoA1 in the additive and dominant models (*p* < 0.001 and *p* = 0.005, respectively) (Table 4). A post hoc power analysis showed that the power for rs117685211 was over 0.9, with similar results observed for the other variants.

### 3.5. Stepwise Linear Regression Analysis for Ox-HDL, ApoA1, and HDL Subfractions

A stepwise linear regression analysis using age, sex, smoking status, and three variants revealed that rs117685211 and rs148877054 genotypes contributed to 0.645% and 0.067% of the variation in serum L-HDL-P and 0.38% and 0.757% in serum M-HDL-P. However, only the rs117685211 genotype contributed to 0.626%, 0.492%, and 0.311% of the variation in ApoA1, HDL-P, and S-HDL-P, respectively (Table 5).

### 3.6. Confirmation of Association of Levels of HDL Subfractions and Ox-HDL with Causal SNPs from Other Population Studies

We evaluated whether genetic variants associated with PAD in populations of European ancestry and Japanese individuals were also associated with PAD in the Taiwanese population. We accordingly detected and confirmed the presence of five PAD-associated causal SNPs in our cohort that were previously reported in Japanese (rs 9584669, rs6842241, and rs2074633) and European (rs12910984 and rs1317286) populations in recent studies. However, our analysis showed that none of them were significantly associated with the levels of Ox-HDL, HDL-P, and S-HDL-P in this study (Table 6).

## 4. Discussion

In the current study, we found that the levels of HDL subfractions and Ox-HDL were strongly associated with the severity of PAD classified according to the Rutherford classification. Furthermore, we identified three SNPs that showed associations with the levels of HDL subfractions and Ox-HDL. To the best of our knowledge, this is the first study reporting an association between the levels of Ox-HDL and HDL subfractions and genetic variants in a Taiwanese PAD population.

Ox-HDL represents a dysfunctional form of HDL formed by oxidative modifications of lipid and protein components in HDL particles following oxidative stress [17,30]. Increased plasma levels of Ox-HDL have been reported in several diseases, such as coronary artery calcification [31], metabolic disease [32], hyperlipidemia [33], and cardiovascular disease [34]. However, there have been no reports on its association with PAD. Our study showed that the levels of Ox-HDL were significantly increased with the severity of PAD classified according to the Rutherford classification. This observation was consistent with previous studies on CVD and metabolic diseases [32,34] and might be attributed to the hydroxyl peroxidation and loss of antioxidant and atheroprotective functions of HDL.

In contrast to Ox-HDL, the effects of HDL particle subfractions in diseases have been controversial; HDL particles are diverse in components, size, and antioxidative activity [10,35]. Moreover, HDL particles have been reported to have different associations with T2D. [36,37]. Previous studies have shown that L and S-HDL-P correlated differently with lipid transport and antioxidative functions [10,38,39,40]. However, studies on the association of M and S-HDL-Ps with T2D have been inconsistent [14,41,42,43,44,45,46].

Our findings of lower levels of S-HDL-P in the group with critical limb ischemia in PAD were consistent with those of Zierfuss et al., who reported that there was a significant inverse association between the levels of S-HDL-P and M-HDL-P and all-cause mortality in statin-treated patients with PAD [47]. In addition, Otvos et al. found that increasing levels of HDL particles (10%) and S-HDL-P (21%) were protective against coronary heart disease events when gemfibrozil was tested in patients with low levels of HDL-C [48]. It is unclear which mechanisms are responsible for these differential associations between HDL subfractions and PAD; however, they might be attributed to the potential antioxidative function of S-HDL-P. The antioxidative activity of HDL subfractions has been shown to increase with particle density, with small HDL exhibiting greater antioxidative capacity than large HDL [49]. Paraoxonase 1, primarily carried by HDL, is a glycoprotein with anti-atherogenic and antioxidant properties [50], predominantly associated with smaller, dense HDL particles where its concentration and enzymatic activity are higher than in larger HDL particles [51,52], and it contributes to atheroprotection in part by promoting cholesterol efflux from macrophages [53]. Besides the aforementioned mechanisms, accumulating evidence indicates that small, dense HDL particles are the most effective mediators of cholesterol efflux [54,55].

Our study found that ApoA1 and S-HDL-P shared similar association patterns with both Rutherford classification and candidate SNPs in patients with PAD. ApoA1, the major protein component of HDL, is primarily responsible for HDL’s antioxidant, anti-inflammatory, and anti-tumorigenic properties [56,57]. The interaction between ApoA1 and ATP-binding cassette transporter A1 mediates cholesterol efflux, a key step in reverse cholesterol transport [58]. Notably, more molecules of ApoA1 are found in smaller HDL particles compared to larger ones, which may enhance their functional capacity [59]. As a result, ApoA1 likely plays a critical role in the protective effects of S-HDL-P in patients with PAD.

In addition, we identified three SNPs that were differentially associated with the levels of Ox-HDL and HDL particles. More specifically, rs117685211 and rs7934858 exhibited opposing associations with the level of Ox-HDL. The clinical features of PAD involve atherosclerotic changes in medium to small arteries. As HDL functions in reducing the accumulation of plaque in the intima of blood vessel, HDL might be the key in preventing disease progression [22]. However, Ox-HDL might alter the antiatherosclerotic function of HDL. Thus, SNPs might reflect the antiatherosclerotic ability of an individual. Furthermore, rs7934858 was found to be located in the 5′ flanking region of LOC105376626 on chromosome 11, and Sultan et al. reported that it might be involved in the regulation of mitochondrial dysfunction, promoting the accumulation of reactive oxygen species (ROS) and leading to endothelial dysfunction [60]. In addition, rs117685211 and rs148877054, both of which are Asian allele-specific SNPs, were associated with lower levels of HDL subclasses. Especially, rs148877054, which is located in the 5′ flanking region of the *LRRK2* gene, was significantly associated with the levels of M and S-HDL-P. Interestingly, Lee et al. reported that *LRRK2* was involved in the regulation of the levels of total cholesterol and triglycerides in hyperlipidemia [61]. Rs117685211 is located on chromosome 1, near the SYT6 gene, which encodes synaptotagmin-6 (SYT6), a member of the synaptotagmin family known for regulating calcium-dependent membrane fusion events [62]. Although the direct role of SYT6 in cholesterol metabolism is unclear, one of the synaptotagmin family members, SYT1, has been shown to alter the hepatic cholesterol content and contribute to hepatic management of hepatic lipids [63]. In addition to the aforementioned findings, the association of rs148877054 genotypes with L-HDL-P is partially suppressed by rs117685211, with the association becoming stronger after adjustment for rs117685211 (Table 5). This may suggest potential interaction and mediation effect between the two SNPs. In the current study, we observed an association between causal SNPs and the levels of Ox-HDL-C in patients with PAD. We also found an association between SNPs and the number of HDL-particle traits. Therefore, we suggested that these minor alleles are associated with the atherogenic phenotype in both Ox-HDL and HDL subfractions, pointing to a potential novel mechanism through which HDL particles affect PAD.

Overall, the novelty of this study was that we found that the patients with PAD in the group of critical limb ischemia were characterized by higher levels of Ox-HDL, whereas they had lower levels of S-HDL-P than those in the group with severe claudication. This phenomenon might be explained by the dysfunction of HDL due to lipid peroxidation and the loss of the antioxidative activity of Ox-HDL. The potential protective effect of S-HDL-P might stem from an enhanced antioxidative function that is modulated in a density-dependent manner. In addition, several causal SNPs, especially an Asian-specific allele, were discovered in our study, which might be helpful in future functional studies and studies exploring drug-targeting mechanisms for the improved treatment of PAD and prevention of disease progression.

This study had several limitations. First, although our findings are tentative, the sample size of patients was relatively small. However, various studies on diverse populations [21,22,23,24], and several studies on the association of the levels of M and S-HDL-P with T2D that were conducted in populations of varying ethnicities, were also based on a limited sample size [14,44,45,46]. We believe that our findings on the relationship between HDL structure–function and PAD are particularly important, especially in the Taiwanese population, because the causal SNPs identified here indicated considerable differences in allele frequencies between Han Chinese and European and American populations, suggesting ethnic specificity. Second, our genetic association study did not include any functional analyses, such as reverse cholesterol transport or cholesterol efflux assays. Therefore, a larger sample size with a Mendelian randomization approach might help to elucidate whether a causal association exists between the levels of HDL subfractions and Ox-HDL and PAD. Another limitation was the lack of a case/control or other ethnic cohort for the clinical application of our results.

## 5. Conclusions

In conclusion, the present study clearly indicated the significance of HDL subfractions and Ox-HDL in disease progression in PAD, although the mechanism involved was not identified. To our knowledge, this is the first study to identify genetic risk factors and loci for PAD in a Taiwanese population. The causal SNPs identified in our study may provide valuable insights for future functional investigations and the development of drug-targeting strategies, potentially enhancing the treatment of PAD and preventing disease progression. We believe that knowledge of the relative molecular pathway and mechanism associated with PAD will contribute to the stratification of patients for the early detection of disease, improved treatment, and potential prevention of disease progression in PAD.

## Figures and Tables

**Table 1:** Clinical and biochemical characteristics of the study population.

	Total	Group I	Group II	*p*
Number	80	19	61	
Sex % (male/female)	57.5/42.5	73.7/26.3	52.5/47.5	0.084
Age (years)	71.52 ± 10.78	69.79 ± 11.06	72.06 ± 10.73	0.426
BMI (kg/m^2^)	24.08 ± 3.74	23.89 ± 3.97	24.14 ± 3. 70	0.804
Current smokers (%)	33.8	63.2	24.6	0.003
Diabetes mellitus (%)	75.0	78.9	73.8	0.451
Hypertension (%)	85.0	89.5	83.6	0.417
HDL-C (mg/dL)	40.23 ± 14.40	46.72 ± 13.57	38.21 ± 12.80	0.018
ApoA1 (g/L)	1.1 ± 0.26	1.23 ± 0.18	1.06 ± 0.27	0.036
LDL-C (mg/dL)	94.19 ± 30.57	101.39 ± 28.63	91.96 ± 31.05	0.256
TG (mg/dL) cholesterol (mg/dL)	138.84 ± 96.44 158.96 ± 37.44	115.83 ± 73.45 169.22 ± 32.70	145.98 ± 102.03 155.78 ± 38.49	0.249 0.185
CRP level (mg/L)	2.65 ± 5.17	0.62 ± 0.51	3.26 ± 5.76	0.058
Rutherford classification				
3 (N = 19)	23.8%			
4 (N = 21)	26.3%			
5 (N = 30)	37.5%			
6 (N = 10)	12.5%			

BMI, body mass index; HDL-C, high-density lipoprotein cholesterol; ApoA1, Apolipoprotein A1; LDL-C, low-density lipoprotein cholesterol; TG, triglycerides. Continuous variables are presented as mean ± SD. Values of lipid profile, ApoA1, and CRP were logarithmically transformed before statistical testing to meet the assumption of normal distributions; however, the untransformed data are shown. Group I: Rutherford grade 3: severe claudication; Group II: Rutherford grade ≧ 4: critical limb ischemia.

**Table 2:** Association of Rutherford classification with Ox-HDL and HDL subfractions.

	Rutherford Classification					Group I	Group II	
	3	4	5	6	*p*	Rutherford Grade 3	Rutherford Grade ≥ 4	*p*
	N = 19	N = 21	N = 30	N = 10		N = 19	N = 61	
Ox-HDL (µg/L)	553.01 ± 138.5	679.81 ± 172.15	823.05 ± 275.11	992.4 ± 288.72	0.001	553.01 ± 138.5	895.11 ± 182.23	0.001
HDL-C (mg/dL)	46.72 ± 13.57	38.43 ± 10.14	38.99 ± 15.51	35.60 ± 9.50	0.109	46.72 ± 13.57	38.21 ± 12.80	0.018
ApoA1 (g/L)	1.23 ± 0.18	1.1 ± 0.25	1.03 ± 0.3	1.05 ± 0.25	0.089	1.23 ± 0.18	1.06 ± 0.27	0.036
Cholesterol (mg/dL)	169.22 ± 32.70	153.05 ± 38.31	153.07 ± 38.99	168.80 ± 38.71	0.362	169.22 ± 32.70	155.78 ± 38.49	0.185
TG (mg/dL)	115.83 ± 73.45	159.1 ± 151.61	131.39 ± 65.04	160.60 ± 58.54	0.468	115.83 ± 73.45	145.98 ± 102.03	0.249
LDL-C (mg/dL)	101.39 ± 28.63	86.50 ± 32.37	91.42 ± 29.49	104.40 ± 32.31	0.311	101.39 ± 28.63	91.96 ± 31.05	0.256
HDL-P (mg/dL)	13.2 ± 1.5	10.5 ± 1.6	10.8 ± 2.3	9.8 ± 5.3	0.055	13.2 ± 1.5	10.1 ± 2.5	0.012
L-HDL-P (mg/dL)	1.2 ± 0.81	0.77 ± 0.58	0.85 ± 0.12	0.86 ± 0.17	0.328	1.2 ± 0.81	0.83 ± 0.11	0.32
M-HDL-P (mg/dL)	3.1 ± 0.25	2.2 ± 1.3	2.1 ± 1.5	2.2 ± 1.8	0.205	3.1 ± 0.25	2.2 ± 1.5	0.187
S-HDL-P (mg/dL)	8.9 ± 2.1	7.5 ± 1.5	7.1 ± 1.2	7.0 ± 1.2	0.052	8.9 ± 2.1	7.1 ± 1.3	0.01

Ox-HDL, oxidized HDL; HDL-C, high-density lipoprotein cholesterol; ApoA1, Apolipoprotein A1; LDL-C, low-density lipoprotein cholesterol; TG, triglycerides; HDL-P, total HDL particle; L-HDL-P, large HDL particle; M-HDL-P, medium HDL particle; S-HDL-P, small HDL particle. Continuous variables are presented as mean ± SD. Values were logarithmically transformed before statistical testing to meet the assumption of normal distributions; however, the untransformed data are shown. Group I: Rutherford grade 3: severe claudication; Group II: Rutherford grade ≥ 4: critical limb ischemia.

**Table 3:** Pearson correlation analysis of Ox-HDL.

	R	*p*
HDL-C (mg/dL)	−0.465	0.001
ApoA1 (g/L)	−0.451	0.005
HDL-P (mg/dL)	−0.483	0.002
L-HDL-P (mg/dL)	−0.378	0.105
M-HDL-P (mg/dL)	−0.382	0.107
S-HDL-P (mg/dL)	−0.402	0.012

HDL-C, high-density lipoprotein cholesterol; ApoA1, Apolipoprotein A1; HDL-P, total HDL particle; L-HDL-P, large HDL particle; M-HDL-P, medium HDL particle; S-HDL-P, small HDL particle.

**Table 4:** Analysis of the association of HDL-C and Ox-HDL levels with causal SNPs.

	MM (%)	Mm (%)	Mm (%)	*p*	MM (%)	Mm + mm (%)	*p*
rs117685211	GG (87.5%)	GT (10%)	TT (2.5%)		GG (87.5%)	GT + TT (12.5%)	
Ox-HDL (µg/L)	621.33 ± 207.02	1103.02 ± 158.22	1117.02 ± 132.05	0.012	621.33 ± 207.02	1109.21 ± 117.33	0.002
ApoA1 (g/L)	1.16 ± 0.2	0.72 ± 0.19	0.41 ± 0.12	1.22 × 10^−8^	1.16 ± 0.2	0.65 ± 0.22	9.23 × 10^−7^
HDL-P (mg/dL)	13.2 ± 2.1	7.1 ± 2.3	4.1 ± 1.1	1.23 × 10^−8^	13.2 ± 2.1	6.2 ± 1.2	1.01 × 10^−6^
L-HDL-P (mg/dL)	1.3 ± 0.52	0.52 ± 0.27	0.12 ± 0.11	2.1 × 10^−4^	1.3 ± 0.52	0.42 ± 0.13	0.001
M-HDL-P (mg/dL)	2.9 ± 0.17	0.93 ± 0.81	0.51 ± 0.25	4.52 × 10^−8^	2.9 ± 0.17	0.82 ± 0.21	0.001
S-HDL-P (mg/dL)	8.5 ± 2.2	5.5 ± 1.2	4.1 ± 1.5	3.82 × 10^−6^	8.5 ± 2.2	4.9 ± 1.27	2.5 × 10^−5^
rs7934858	TT (60%)	TC (37.5%)	CC (2.5%)		TT (60%)	TC + CC (40%)	
Ox-HDL (µg/L)	791.01 ± 233.12	583.27 ± 209.32	1127.5 ± 211.56	0.001	791.01 ± 233.12	604.21 ± 276.3	0.002
ApoA1 (g/L)	1.11 ± 0.21	1.14 ± 0.33	0.68 ± 0.22	0.196	1.11 ± 0.21	1.11 ± 0.33	0.196
HDL-P (mg/dL)	13.5 ± 1.3	12.3 ± 2.5	6.2 ± 1.1	0.284	13.5 ± 1.3	12.5 ± 2.1	0.423
L-HDL-P (mg/dL)	1.22 ± 1.55	1.2 ± 0.27	11 ± 0.88	0.55	1.22 ± 1.55	1.4 ± 0.72	0.455
M-HDL-P (mg/dL)	2.4 ± 1.22	2.2 ± 0.7	0.91 ± 0.83	0.624	2.4 ± 1.22	2.3 ± 1.2	0.832
S-HDL-P (mg/dL)	7.7 ± 0.5	7.6 ± 1.8	6.2 ± 1.1	0.872	7.7 ± 0.5	7.8 ± 3.5	0.851
rs148877054	GG (93.5%)	GT (4%)	TT (2.5%)		GG (93.5%)	GT + TT (6.5%)	
Ox-HDL (µg/L)	854.14 ± 211.32	901.22 ± 137.25	1023.12 ± 127.34	0.133	854.14 ± 211.32	977.37 ± 275.22	0.131
ApoA1 (g/L)	1.12 ± 0.24	1.2 ± 0.23	0.41 ± 0.11	4.3 × 10^−4^	1.12 ± 0.24	0.7 ± 0.42	0.005
HDL-P (mg/dL)	12.1 ± 2.5	7.3 ± 1.2	3.9 ± 1.2	1.5 × 10^−4^	12.1 ± 2.5	6.5 ± 1.3	0.001
L-HDL-P (mg/dL)	1.2 ± 0.23	1.2 ± 0.33	1.1 ± 0.72	0.077	1.2 ± 0.23	1.1 ± 0.21	0.328
M-HDL-P (mg/dL)	2.5 ± 1.3	2.2 ± 0.8	0.82 ± 0.13	1.37 × 10^−11^	2.5 ± 1.3	1.3 ± 1.2	1.01 × 10^−5^
S-HDL-P (mg/dL)	7.4 ± 1.2	5.8 ± 1.5	4.3 ± 1.7	1.1 × 10^−4^	7.4 ± 1.2	4.5 ± 1.5	1.1 × 10^−4^

MM homozygosity of major allele, Mm heterozygosity, mm homozygosity of minor allele; Ox-HDL, oxidized HDL; ApoA1, Apolipoprotein A1; HDL-P, total HDL particle; L-HDL-P, large HDL particle; M-HDL-P, medium HDL particle; S-HDL-P, small HDL particle. Continuous variables are presented as mean ± SD. Values were logarithmically transformed before statistical testing to meet the assumption of normal distributions; however, the untransformed data are shown.

**Table 5:** Ox-HDL, ApoA1, and HDL subfractions: stepwise linear regression analysis, including genotypes.

	Ox-HDL			ApoA1			HDL-P			L-HDL-P			M-HDL-P			S-HDL-P		
	β	r^2^	*p*	β	r^2^	*p*	β	r^2^	*p*	β	r^2^	*p*	β	r^2^	*p*	β	r^2^	*p*
rs117685211	0.157	0.156	0.009	−0.225	0.626	4.5 × 10^−10^	−0.222	0.492	1.61 × 10^−7^	−1.003	0.645	0.001	−0.726	0.38	0.01	−1.31	0.311	2.0 × 10^−4^
rs7934858	−0.113	0.117	0.023	0.075	-	0.446	−0.151	-	0.179	−0.022	-	0.817	−0.91	-	0.243	−0.18	-	0.169
rs148877054	−0.187	-	0.514	0.225	-	0.149	0.182	-	0.313	−1.065	0.067	0.004	−1.779	0.757	2.84 × 10^−6^	0.044	-	0.835

Ox-HDL, oxidized HDL; ApoA1, Apolipoprotein A1; HDL-P, total HDL particle; L-HDL-P, large HDL particle; M-HDL-P, medium HDL particle; S-HDL-P, small HDL particle.

**Table 6:** Analysis of the correlation of levels of Ox-HDL, HDL-P, and S-HDL-P with PAD-associated causal SNPs from other population studies.

Gene SNP	Genotypes (%)	Ox-HDL (µg/L)	*p*	S-HDL-P (mg/dL)	*p*	HDL-P (mg/dL)	*p*
rs9584669 *	AA (81.2)	245.12 ± 281.43	0.243	7.5 ± 3.1	0.917	11.1 ± 1.8	0.873
	AG (18.8)	628.12 ± 147.22		7.8 ± 2.3		11.9 ± 1.5	
rs6842241 *	AA (55)	655.13 ± 234.27	0.137	7.7 ± 3.2	0.256	11.3 ± 1.8	0.184
	AG (40)	811.32 ± 245.12		7.1 ± 1.5		11.3 ± 2.5	
	GG (5)	587.55 ± 232.18		8.4 ± 0.23		11.5 ± 0.08	
	AA (55)	655.13 ± 234.27	0.22	7.7 ± 3.2	0.343	11.3 ± 1.8	0.238
	AG + GG (45)	781.24 ± 252.17		7.7 ± 1.8		10.8 ± 3.1	
rs2074633 *	AA (28.7)	807.25 ± 287.18	0.123	7.1 ± 1.4	0.723	11.4 ± 2.8	0.903
	AG (57.5)	644.23 ± 217.19		8.1 ± 1.3		11.1 ± 2.5	
	GG (13.8)	778.22 ± 260.25		6.9 ± 2.5		9.8 ± 3.5	
	AA (28.7)	807.25 ± 287.18	0.216	7.1 ± 1.4	0.314	11.4 ± 2.8	0.599
	AG + GG (7.13)	688.34 ± 247.12		7.5 ± 1.5		11.4 ± 2.2	
rs12910984 ^#^	AA (28.7)	652.81 ± 241.34	0.324	7.9 ± 1.7	0.903	11.8 ± 2.1	0.602
	AG (56.3)	742.32 ± 260.32		7.8 ± 1.3		10.5 ± 3.8	
	GG (15)	755.18 ± 314.25		8.1 ± 0.6		12.5 ± 1.7	
	AA (28.7)	652.81 ± 241.34	0.237	7.9 ± 1.7	0.526	11.8 ± 2.1	0.578
	AG + GG (71.3)	762.54 ± 238.24		7.7 ± 1.2		10.8 ± 2.6	
rs1317286 ^#^	AA (81.2)	741.34 ± 251.14	0.521	7.5 ± 1.2	0.812	11.8 ± 2.5	0.586
	AG (16.3)	708.17 ± 232.43		7.7 ± 1.5		11.5 ± 2.8	
	GG (2.5)	434.18 ± 258.17		6.9 ± 1.2		9.1 ± 1.2	
	AA (81.2)	741.34 ± 251.14	0.413	7.5 ± 1.7	0.938	11.8 ± 2.5	0.604
	AG + GG (18.8)	687.15 ± 265.38		7.5 ± 2.5		11.1 ± 2.8	

* indicates the causal SNPs from a Japanese population study; ^#^ indicates the causal SNPs from a European population study. Ox-HDL, oxidized HDL; HDL-P, total HDL particle; S-HDL-P, small HDL particle. Continuous variables are presented as mean ± SD. Values were logarithmically transformed before statistical testing to meet the assumption of normal distributions; however, the untransformed data are shown.

## Data Availability

The original contributions presented in this study are included in the article/Supplementary Material. Further inquiries can be directed to the corresponding author(s).

## Supplementary Materials

The following supporting information can be downloaded at: https://www.mdpi.com/article/10.3390/biom15101419/s1, Table S1: Summary of the study variants.

## Abbreviations

The following abbreviations are used in this manuscript:

HDL	High-density lipoprotein
HDL-P	High-density lipoprotein particle
HDL-C	High density lipoprotein cholesterol
L-HDL-P	Large high-density lipoprotein particle
M-HDL-P	Medium high-density lipoprotein particle
S-HDL-P	Small high-density lipoprotein particle
Ox-HDL	Oxidized high-density lipoprotein
GWAS	Genome-wide association studies
SNP	Single nucleotide polymorphism
PAD	Peripheral artery disease
CVD	Cardiovascular disease
RCT	Reverse cholesterol transport
LDL-C	Low-density lipoprotein cholesterol
NMR	Nuclear magnetic resonance
CHD	Coronary heart disease
NO	Nitric oxide
MPO	Myeloperoxidase
T2D	Type 2 diabetes
EVI	Endovascular intervention
CLI	Critical limb ischemia
PBMCs	Peripheral blood mononuclear cells
RBC	Red blood cell
BMI	Body mass index
TG	Triglycerides
ROS	Reactive oxygen species
CML	Carboxymethyl lysine
SYT	Synaptotagmin
ApoA1	Apolipoprotein A1
