# Supplementary material for: Effects of HDL Structure and Function in Peripheral Artery Disease
Source: Biomolecules. 2025 Oct 6;15(10):1419. doi: 10.3390/biom15101419 (PMC12564848; doi:10.3390/biom15101419)
Supplement: Supplementary file 1 [file biomolecules-15-01419-s001.zip › biomolecules-3852394-supplementary.pdf]

Supplementary Table S1. Summary of the study variants

SNP number	Chromosome	Position	Location	Minor allele	MAF	HWE
rs117685211	1	114087810	Near *SYT6* gene	T	0.075	0.658
rs7934858	11	35037130	Near LOC105376626	C	0.213	0.819
rs148877054	12	40288195	Near *LRRK2* gene	T	0.045	0.163

MAF: minor allele frequency; HWE: Hardy–Weinberg equilibrium
